# Imaging Modalities for Differentiating Bone Infarction and Osteomyelitis in Sickle Cell Disease: A Literature Review

**DOI:** 10.7759/cureus.90096

**Published:** 2025-08-14

**Authors:** Ali M Juma, Qasim M Shamtoot, Maawa M Juma

**Affiliations:** 1 Medicine, Salmaniya Medical Complex, Manama, BHR; 2 Emergency Medicine, Salmaniya Medical Complex, Manama, BHR

**Keywords:** bone infarction, magnetic resonance imaging, osteomyelitis, plain radiography, radionuclide imaging, sickle cell disease, ultrasonography, vaso-occlusive crisis

## Abstract

Sickle cell disease (SCD) is a hereditary hemoglobinopathy associated with frequent acute and chronic skeletal complications. Among these, vaso-occlusive crises (VOCs)-induced bone infarctions and osteomyelitis are two critical yet clinically overlapping conditions that pose a diagnostic challenge. Accurate differentiation between the two is essential, as they require markedly different treatment strategies, conservative management for VOCs versus antibiotic therapy, and possible surgical intervention for osteomyelitis. This comparative review explores various imaging modalities’ roles, advantages, and limitations, including plain radiography, ultrasonography, magnetic resonance imaging (MRI), and radionuclide scans, such as bone and labeled leukocyte imaging. While no single modality has emerged as the gold standard, MRI and combined radionuclide approaches demonstrate promising sensitivity and specificity when interpreted alongside clinical and laboratory findings. This paper also highlights the limitations of various imaging techniques, underscores the necessity of correlating imaging findings with clinical data, and addresses the risk of misdiagnosis. A comprehensive understanding of the imaging characteristics of these complications is crucial for orthopedic surgeons, hematologists, radiologists, and infectious disease specialists involved in the care of patients with SCD. This review emphasizes a multimodal diagnostic approach tailored to the clinical context, aiming to guide clinicians in optimizing patient care for individuals with SCD presenting with acute bone pain.

## Introduction and background

Background information

Sickle cell disease (SCD) is a clinically significant hereditary hemoglobinopathy that has followed an increasing trend worldwide. SCD patients are at risk of different acute and chronic systemic complications. Bone involvement is a significant complication associated with SCD, ranging from acute complications, such as vaso-occlusive crises (VOCs) and osteomyelitis, to chronic complications, such as osteopenia, osteoporosis, and osteonecrosis. The most typical presenting complaint of SCD patients that results in hospitalization is acute severe bone pain that could be a result of acute bone infarction resulting from either a VOC or an osteomyelitis superimposed bacterial infection [1,2].

The most frequent clinical presentation of SCD is VOCs, defined as microvascular obstruction of post-capillary venules by sickled red blood cells (RBCs), resulting in acute tissue infarction, chronic tissue infarction, hemolysis, erythrocyte membrane damage, and acute painful crises [3]. The combined effects of VOC-induced tissue infarction, elevated iron levels, and splenic dysfunction-induced immunodeficiency increase the risk of osteomyelitis [4]. Moreover, recurrent VOCs can result in chronic vascular insufficiency and anatomical deformities, which may predispose individuals to osteomyelitis, particularly due to hematogenous spread of bacteria, most commonly Salmonella species [4-6]. Therefore, acute bone infarctions and osteomyelitis should be seen in association, and their overlapping clinical features necessitate careful evaluation through imaging to aid in accurate diagnosis and management [7].

Epidemiological relevance

Global data reports that 5.2% of SCD patients develop three to 10 acute bone crises that could require up to three hospitalizations annually [8]. However, most patients’ pain resolves within five to seven days. Rarely, severe crises may persist for weeks or months [9].

In endemic areas like the Kingdom of Bahrain, acute bone crises account for 25% of hospital budgets. In 1995, according to Arrayed and Haites, Bahrain had a local population of 558,879, with 1%, approximately 5,589 individuals, diagnosed with SCD [10,11]. Since then, the country’s population has grown to around 1.5 million. As of March 2024, the Supreme Council of Health reported that around 7,000 people were living with SCD, accounting for roughly 0.47% of the total population [12]. While this marks a decline in the overall percentage, it does not suggest that SCD is nearing eradication, as the absolute number of affected individuals continues to increase. Furthermore, several factors must be considered, including the rise in foreign residents, environmental changes, the expansion of premarital screening programs, and advancements in awareness and counseling services [2].

Overlap between vaso-occlusive crises, induced bone infarction, and osteomyelitis

Acute bone infarction has a 50-fold higher incidence than osteomyelitis in SCD patients [13,14]. It is particularly challenging to distinguish between VOCs and osteomyelitis in the acute phase, as both complications can present with fever, pain, swelling, limb tenderness, limited range of motion, elevated inflammatory markers, leukocytosis, and normal blood cultures in the early stages (Table 1) [6,13-15].

**Table 1:** Distinguishing clinical features and findings between vaso-occlusive crisis and osteomyelitis. This table was taken from an open-access article distributed under the Creative Commons Attribution License 4.0 (CCBY 4.0) [16].

Factor	Vaso-occlusive crises-induced bone infarction	Osteomyelitis
Prevalence	50 times more common than osteomyelitis	-
History and physical examination		
Fever	More than 38°C fever is possible	More than 38°C fever is more likely to be present 24 hours before the initial presentation
Location of pain	Multiple sites of pain may be present	More likely to cause pain localized to a single area, most commonly involving the diaphysis of a long bone
Joint appearance	Swelling is possible	Swelling more likely
Laboratory testing		
Leukocytes	Normal to mild elevation	Elevation may be present
Inflammatory markers (C-reactive protein, erythrocyte sedimentation rate)	Normal to mild elevation	Not specific to osteomyelitis, more pronounced elevations are typically observed in osteomyelitis compared to infarction
Cultures	-	The gold standard diagnosis is a positive culture from blood, bone, or synovial fluid

While there is a significant overlap in the clinical and laboratory features, the gold standard for the diagnosis of osteomyelitis is a positive blood, bone, or synovial fluid culture; however, this is often not feasible due to the invasiveness of the procedure [14,17]. This complexity in the diagnostic process leads to delaying the treatment plan, increases the rates of hospitalizations, and unnecessary regimens that lead to further complications (e.g., chronic osteomyelitis and joint destruction), especially in the pediatric population (most affected population) [4]. The treatment algorithms for VOCs and osteomyelitis differ significantly, with VOCs primarily treated conservatively with analgesia, hydration, and supportive care for several days, while osteomyelitis mainly requires targeted antibiotics for weeks, and surgical drainage if indicated [13,15]. As such, accurate differentiation between the two is of utmost importance.

The reliance and integration of radiological investigations in differentiating between bone infarction and osteomyelitis is owed to the recent developments and innovations in radiology that enhanced the visualization of bone involvement in SCD. A variety of imaging modalities, such as plain radiography (X-ray), ultrasonography, magnetic resonance imaging (MRI), and radionuclide imaging (bone and labeled leukocytes scans), are utilized in the diagnostic workup. Each of these modalities has its strengths and limitations regarding its specificity, sensitivity, cost, ability to detect early changes, and availability. However, no consensus exists in the medical community regarding the gold standard radiological modality in diagnosing bone infarction and osteomyelitis [14].

This comparative review article provides an in-depth analysis of multiple imaging modalities currently used to diagnose acute VOCs-induced bone infarctions and osteomyelitis, highlighting their comparative utility, diagnostic features, and new techniques that may transform imaging accuracy. This was accomplished through the analysis of various articles, guidelines, and case reports, with the primary objective of offering clinicians a clear and practical approach to selecting the most appropriate imaging modality for diagnosing complications of SCD.

Methods

Multiple databases were used throughout the paper to ensure a comprehensive and impartial narrative literature review. Neither a systematic review nor a meta-analysis were suitable for this article because of the heterogeneity of the study designs used, different outcome metrics, variability of patient population, limited number of comparable studies for each modality, and inconsistent reporting of data. Relevant articles were sourced from PubMed, Google Scholar, ScienceDirect, and Embase. The cited literature spans publications from January 1976 to May 2025 and includes only papers published in English. The search strategy involved a combination of the following keywords: sickle cell disease, sickle cell anemia, vaso-occlusive crisis, bone infarction, osteomyelitis, imaging modalities, plain radiography, ultrasonography, MRI, radionuclide imaging, hematology, infection, and radiology. The “Medscape” website was frequently used for up-to-date diagnostic guidelines and statistical data. In certain areas where peer-reviewed literature was limited, non-peer-reviewed sources were also included. Additional articles and papers from various journals were externally searched to broaden the scope of analysis. The review incorporates many study types, including case reports and experimental research. In total, 43 journal articles, four online resources, and one book were reviewed.

The inclusion criteria for this review required that selected articles be published in English and be accessible as open-access texts. Additionally, only studies that provided a clear and detailed description of their methodology and results were considered for inclusion. These criteria ensured that the selected literature was both comprehensible and sufficiently robust for critical analysis.

Conversely, studies were excluded if they fell into the category of editorials, commentaries, or opinion pieces, as these formats typically lack primary research data and rigorous methodology. Articles were also excluded if the full text was not available or if the research was still in progress or incomplete, as these would not provide the necessary information for comprehensive evaluation and comparison.

## Review

Plain radiography (X-ray)

In orthopedic surgery and emergency medicine, plain radiographs play a crucial role as the most used first-line imaging modality due to their cost-effectiveness, relative safety, and availability. In acute VOC-induced bone infarctions, however, plain radiographs are not helpful in the diagnostic process as they are mostly normal in the acute phase of bone infarction. In the ensuing months following the bone infarction, plain radiographs may show areas of ill-defined translucency, followed by arc-like subchondral patchy sclerosis, periosteal reactions, and intramedullary mottled lucent areas (strand-like increased opacities in the medullary areas) [14]. These changes typically occur in the metaphyseal areas of the long bones [18,19].

Occasionally, distinctive radiographic features may be observed that are specific to bone infarction. When the central portion of a vertebral endplate infarcts, it results in a characteristic “H-deformity,” and when this angular depression appears at multiple vertebral levels, it is considered pathognomonic for vaso-occlusive crisis (VOC)-induced bone infarctions [20]. Infarction of the epiphyseal region can lead to premature fusion or a cone-shaped epiphysis, often resulting in shortening of the affected bone [20]. In cases of multiple bony infarctions in long bones, sclerosis may manifest as a “bone within a bone” appearance, representing an old cortex within a new cortex [20]. Hand-foot syndrome (dactylitis) may present radiographically as patchy lucent areas accompanied by soft tissue swelling and periostitis of the metacarpal or metatarsal bones [21]. Additional vertebral signs that may indicate bone infarction include vanishing vertebrae, tower vertebrae, and anterior vascular notches [22].

Similarly, the radiographs of the early stages of osteomyelitis are usually normal. In some cases, osteomyelitis could present as osteopenia and periostitis as non-specific changes that may be seen with associated soft tissue swelling and aggressive bone destruction [14,23,24]. However, similar findings may also be observed in bone infarctions, thus limiting their clinical value [19]. Lytic lesions, typically suggestive of osteomyelitis, only appear two weeks after the initial infection (lucent areas appear only in the later stages of the disease’s natural progression) [23,24]. The poor sensitivity and specificity of plain radiography in diagnosing acute VOC-induced bone infarction and osteomyelitis require using other imaging modalities in conjunction with the clinical picture [23,25].

Ultrasonography

Although the role of ultrasonography in deep bone imaging is limited, it remains a rapid, economically feasible, non-invasive, portable, and user-friendly imaging technique that effectively targets specific areas of pain and is particularly useful in resource-limited areas and the pediatric population [14,26]. Ultrasonography demonstrated a sensitivity of 74% in diagnosing osteomyelitis in SCD. In certain cases, with raised C-reactive protein or raised white cell count, the sensitivity of ultrasonography was reported as high as 76% [14,25,27-30].

In acute osteomyelitis, ultrasonography may show early indirect signs, such as acute extraosseous pathology, periosteal elevation (subperiosteal abscess), and joint effusions [14,27]. However, like CT scans, the primary finding (subperiosteal fluid) can also be seen in bone infarction caused by VOCs, typically with less depth than osteomyelitis, thus limiting its diagnostic specificity [14,31]. Some papers reported that fluid levels more than 4 mm in depth entail a high clinical suspicion of osteomyelitis [27].

In a retrospective study from Saudi Arabia, ultrasonography accurately identified 17 patients with acute osteomyelitis from 53 patients with SCD and VOCs. This study’s authors recommended that ultrasonography can be useful for differentiating acute osteomyelitis from VOCs in certain cases [17,29]. Another study reported that it had less success differentiating the two using ultrasonography, with a significant percentage of false negative results [17,32]. These contradictions and inconsistencies in the reported accuracy of ultrasonography may be attributed to its user-dependent nature and the need for a high level of operator expertise. To address these limitations, it is recommended that ultrasonography be interpreted alongside the clinical presentation, culture results, and other imaging modalities to accurately distinguish between osteomyelitis and bone infarction.

A retrospective case-control study evaluated the use of ultrasonography compared to other imaging modalities. It is recommended that MRI be the most appropriate next step for patients with normal ultrasonography findings, especially in cases of high clinical suspicion [25].

Magnetic resonance imaging

MRI is a sensitive imaging modality for identifying bone marrow and bone infarction [3,14,33-37]. Ill-defined bone marrow signal changes, enhancing periosteal reaction, and soft tissue abscesses are frequently seen in the first few days of bone infarction [14,36,37]. On low-signal intensity and T1-weighted images, bone infarctions appear as a well-defined serpiginous area surrounded by high-signal intensity on T2-weighted images and short tau inversion recovery (STIR) sequence. This represents marrow edema. With time, the pathological process becomes focal, with T1-weighted images showing a serpentine line of low-signal intensity surrounding a hyper-intense marrow (double line of low intensity surrounding inner high-signal intensity might be seen in T2-weighted images as well) [20,23,38].

However, as with other imaging modalities, these acute findings are like those in acute osteomyelitis and are thus difficult to distinguish. Therefore, some guidelines have recommended that MRI should not be routinely used in managing bone infarctions and should be reserved for diagnosing patients with persistent symptoms despite supportive management or when there is high clinical suspicion of acute osteomyelitis [14].

Like other imaging modalities, bone infarction and acute osteomyelitis often exhibit overlapping radiological features. In both complications, MRI would show reactive marrow edema (detectable as early as 24 hours post-infection in acute osteomyelitis) with surrounding hyperemia [14,24,36,39]. Specific features for osteomyelitis involve soft tissue collections and the replacement of the normal high-signal intensity of fatty marrow on T1-weighted images with low-signal intensity, indicating infection. These areas typically appear as regions of increased signal intensity on T2-weighted images due to bone destruction [20,40]. A recent study involving nine patients with SCD concluded that when gadolinium enhancement is utilized, the diagnostic accuracy increases significantly, thereby aiding in identifying areas of infection [17,39].

Several papers have recommended dynamic enhanced-contrast MRI and diffusion-weighted imaging to increase the specificity of the results. In the clinical setting, once osteomyelitis has been diagnosed by the culture results, MRI may be used as a highly specific localization modality of the lesion and to monitor the patient’s response to the antibiotic regimen [14,23,36].

Although MRI is a highly sensitive imaging modality, with reported sensitivity ranging from 82 to 100%, its specificity is relatively lower, ranging from 75 to 96%. As such, clinical correlation is essential when interpreting MRI findings [23,41]. Additionally, MRI may overestimate the extent of infection, and its findings can often overlap with those of other conditions, such as malignancies [23,42]. Furthermore, MRI is not cost-effective and may be unsuitable for certain patient populations, including children, individuals with metal implants, and cases requiring imaging of large anatomical regions [23,43].

The literature, however, has significant gaps and contradictions regarding the exact role of MRI. While some studies advocate for its use as a diagnostic tool for both complications, others downplay its utility, emphasizing the importance of correlating MRI findings with clinical assessment and other imaging modalities. As such, further research is required to accurately clarify its diagnostic value and establish its effectiveness in clinical practice.

Radionuclide imaging

Bone Scanning

Bone scans reflect reparative osteoblastic response in SCD as isotopes initially localize in the capillaries and surrounding perivascular spaces, followed by accumulation in regions of reactive new bone formation [23,41]. Radioisotope bone scan uses a combination of technetium-99m (99mTc)-diphosphonate (measuring bone uptake) and 99mTc-labeled sulfur colloid (measuring bone marrow uptake). Bone scanning provides a whole-body assessment of the axial and appendicular skeleton, making it possible to identify multiple areas of involvement, thereby allowing it to accurately identify areas of infarction acutely [14,17,44-46].

Among the few distinguishing features between bone infarction and osteomyelitis, bone infarctions typically present as photopenic (cold) areas with circumferential rim enhancement during the acute phase due to vascular compromise. These areas may later evolve into regions of increased uptake during the healing process. On the other hand, acute osteomyelitis can present with increased focal activity with either an increased or a decreased uptake. However, both may show increased uptake hot spots, especially in later stages [17,47]. According to many papers, however, false positives and false negatives still occur, leading to the recommendation of using 99mTc with gallium to improve accuracy. Moreover, a three-phase bone scan improves specificity as it shows persistent hyperemia in all phases in acute osteomyelitis, while an evolving and increased or delayed uptake may suggest bone infarction [14,17,44-46].

Different papers have recommended that, should bone scanning be inconclusive within the first 24 hours of the initial presentation, the combination of sequential bone marrow and bone scans can be advantageous in the differentiation between bone infarctions and osteomyelitis [17]. Despite its high sensitivity, multiple studies have proven bone scans to have a lower specificity, hence retaining a limited role in differentiating bone infarction from osteomyelitis [14,44,45,48]. Therefore, it is not recommended as a routine diagnostic modality in bone infarctions and acute osteomyelitis [14].

Labeled Leukocytes Scans

Indium-111 or 99mTc-labeled leukocyte scans detect the migration of leukocytes to the sites of inflammation, making them more sensitive (90-93%) and specific (85-89%) for infection. A positively labeled leukocyte scan is highly suggestive of acute osteomyelitis, particularly when supported by positive bone scan findings, as this enhances diagnostic precision and anatomical localization [23,41].

The scan has limitations in demonstrating bony involvement and distinguishing between bone and soft tissue inflammation. Therefore, its effectiveness largely depends on correlation with bone scan findings. Moreover, its limited availability in many healthcare facilities and the extended time (48-72 h) required to perform the scan make it unsuitable for routine use in the clinical setting [23,41]. A summarized comparison of diagnostic imaging features distinguishing bone infarction and osteomyelitis across modalities is presented in Table 2 [14,17,20,23-27,31,36-41,43-48].

**Table 2:** Diagnostic imaging modalities: key differentiators between bone infarction and osteomyelitis in sickle cell disease. This table is created by the authors of this study with the help of multiple sources. STIR: short tau inversion recovery

Imaging modalities	Findings seen in vaso-occlusive crises-induced bone infarction	Findings seen in osteomyelitis	Advantages and limitations
X-ray	Initially clear without any abnormality. Patchy lucency, sclerosis, and periosteal reaction were seen later [14].	Osteopenia, periostitis, soft tissue swelling, bone destruction (late) [14,23,24].	Poor sensitivity and specificity, cheap, but useful only in the chronic phase [14,23,25].
Ultrasound	Subperiosteal fluid is typically seen with less depth than osteomyelitis [14,31].	Subperiosteal abscess, joint effusion, fluid >4 mm, hyperemia [14,27].	Poor sensitivity, operator-dependent, cheap, useful in children, and best when combined with labs [14,15,26,27].
MRI	Serpiginous low T1 and high T2/STIR signal; marrow edema with no abscess [14,20,23,36-38].	Vague marrow edema, soft tissue changes, periosteal enhancement, abscess [14,20,24,36,39,40].	Most sensitive, enhanced MRI improves specificity, but is expensive [14,17,23,36,39,41,43].
Bone scan	Cold (photopenic) areas with circumferential rim enhancement in the acute phase, and may show hot in the chronic phase [17,47].	Increased focal activity with either an increased or a decreased uptake. May show increased uptake hot spots, especially in the chronic phase [17,47].	High sensitivity, low specificity, and used in combination with other clinical and investigative parameters [14,44,45,48].
Labeled leukocyte scan	Usually no findings [23,41].	High uptake in infected regions [23,41].	Highly specific for infection, but limited role due to availability and scan time [23,41].

## Conclusions

Distinguishing between VOCs-induced bone infarction and osteomyelitis in SCD remains a diagnostic challenge due to overlapping clinical and imaging features. Accurate and timely diagnosis is crucial, as the management strategies for each condition differ significantly.

While initial imaging with X-ray and ultrasound is useful for screening, more advanced modalities like MRI and radionuclide scans offer greater sensitivity, albeit with limitations in specificity and accessibility. No single imaging technique can reliably distinguish between the two conditions on its own. Therefore, a multimodal approach that integrates clinical assessment, laboratory results, and complementary imaging is essential for improving diagnostic accuracy and guiding treatment decisions. Timely identification and appropriate treatment are critical to preventing complications and improving patient outcomes. Future advances in imaging may enhance specificity, but for now, clinical context remains key in determining the most appropriate diagnostic and therapeutic course.

## References

[1] Hoffbrand AV, Moss PA (2015). Essential Haematology. Seventh Edition. https://www.google.co.in/books/edition/Hoffbrand_s_Essential_Haematology/lwieCAAAQBAJ?hl=en&gbpv=0.

[2] Juma A, Shamtoot Q, Juma MM (2025). The vertebral manifestations of sickle cell disease: a literature review. J Med Health Stud.

[3] Mankad VN, Williams JP, Harpen MD (1990). Magnetic resonance imaging of bone marrow in sickle cell disease: clinical, hematologic, and pathologic correlations. Blood.

[4] Berger E, Saunders N, Wang L, Friedman JN (2009). Sickle cell disease in children: differentiating osteomyelitis from vaso-occlusive crisis. Arch Pediatr Adolesc Med.

[5] Blacksin MF, Finzel KC, Benevenia J (2001). Osteomyelitis originating in and around bone infarcts: giant sequestrum phenomena. AJR Am J Roentgenol.

[6] Piehl FC, Davis RJ, Prugh SI (1993). Osteomyelitis in sickle cell disease. J Pediatr Orthop.

[7] Wong AL, Sakamoto KM, Johnson EE (2001). Differentiating osteomyelitis from bone infarction in sickle cell disease. Pediatr Emerg Care.

[8] Platt OS, Thorington BD, Brambilla DJ, Milner PF, Rosse WF, Vichinsky E, Kinney TR (1991). Pain in sickle cell disease - rates and risk factors. N Engl J Med.

[9] Shapiro BS (1989). The management of pain in sickle cell disease. Pediatr Clin North Am.

[10] Al Arrayed SS, Haites N (2025). Features of sickle-cell disease in Bahrain. East Mediterr Health J.

[11] Bahrain Open Data Portal. (2024 (2025). Population by nationality and sex. https://www.data.gov.bh/explore/dataset/03-population-by-nationality-and-sex/table/?disjunctive.nationality&disjunctive.sex&sort=-year&refine.year=1995.

[12] Al-Shakhouri Al-Shakhouri, M. (2024, March 2 (2025). 7000 people suffer from sickle cell disease in Bahrain. https://www.alayam.com/alayam/first/1047045/News.html.

[13] Keeley K, Buchanan GR (1982). Acute infarction of long bones in children with sickle cell anemia. J Pediatr.

[14] Almeida A, Roberts I (2005). Bone involvement in sickle cell disease. Br J Haematol.

[15] Dalton GP, Drummond DS, Davidson RS, Robertson Jr WW (1996). Bone infarction versus infection in sickle cell disease in children. J Pediatr Orthop.

[16] Al Farii H, Zhou S, Albers A (2020). Management of osteomyelitis in sickle cell disease: review article. J Am Acad Orthop Surg Glob Res Rev.

[17] Skaggs DL, Kim SK, Greene NW, Harris D, Miller JH (2001). Differentiation between bone infarction and acute osteomyelitis in children with sickle-cell disease with use of sequential radionuclide bone-marrow and bone scans. J Bone Joint Surg Am.

[18] Miller RG, Segal JB, Ashar BH (2006). High prevalence and correlates of low bone mineral density in young adults with sickle cell disease. Am J Hematol.

[19] Lonergan GJ, Cline DB, Abbondanzo SL (2001). Sickle cell anemia. Radiographics.

[20] Ramirez Ramirez, I. (2019, July 24 (2025). Sickle cell anemia skeletal imaging. https://emedicine.medscape.com/article/413542-overview.

[21] Espinosa GA (1979). Hand-foot roentgen findings in sickle cell anemia. J Natl Med Assoc.

[22] Stanislavsky A, Yap J, Weerakkody Y (2025). Sickle cell disease (skeletal manifestations). Radiopaedia.

[23] Vanderhave KL, Perkins CA, Scannell B, Brighton BK (2018). Orthopaedic manifestations of sickle cell disease. J Am Acad Orthop Surg.

[24] Calhoun JH, Manring MM (2005). Adult osteomyelitis. Infect Dis Clin North Am.

[25] Inusa BP, Oyewo A, Brokke F, Santhikumaran G, Jogeesvaran KH (2013). Dilemma in differentiating between acute osteomyelitis and bone infarction in children with sickle cell disease: the role of ultrasound. PLoS One.

[26] Sidhu PS, Rich PM (1999). Sonographic detection and characterization of musculoskeletal and subcutaneous tissue abnormalities in sickle cell disease. Br J Radiol.

[27] William RR, Hussein SS, Jeans WD, Wali YA, Lamki ZA (2000). A prospective study of soft-tissue ultrasonography in sickle cell disease patients with suspected osteomyelitis. Clin Radiol.

[28] Rifai A, Nyman R (1997). Scintigraphy and ultrasonography in differentiating osteomyelitis from bone infarction in sickle cell disease. Acta Radiol.

[29] Sadat-Ali M (1998). The status of acute osteomyelitis in sickle cell disease. A 15-year review. Int Surg.

[30] Riebel T, Kebelmann-Betzing C, Götze R, Overberg US (2003). Transcranial Doppler ultrasonography in neurologically asymptomatic children and young adults with sickle cell disease. Eur Radiol.

[31] Stark JE, Glasier CM, Blasier RD, Aronson J, Seibert JJ (1991). Osteomyelitis in children with sickle cell disease: early diagnosis with contrast-enhanced CT. Radiology.

[32] Howard CB, Einhorn M, Dagan R, Nyska M (1993). Ultrasound in diagnosis and management of acute haematogenous osteomyelitis in children. J Bone Joint Surg Br.

[33] Mankad VN, Yang YM, Williams JP, Brogdon BG (1988). Magnetic resonance imaging of bone marrow in sickle cell patients. Am J Pediatr Hematol Oncol.

[34] van Zanten TE, Statius LW, Golding RP, Valk J (1989). Imaging the bone marrow with magnetic resonance during a crisis and in chronic forms of sickle cell disease. Clin Radiol.

[35] Deely DM, Schweitzer ME (1997). MR imaging of bone marrow disorders. Radiol Clin North Am.

[36] Bonnerot V, Sebag G, de Montalembert M, Wioland M, Glorion C, Girot R, Lallemand D (1994). Gadolinium-DOTA enhanced MRI of painful osseous crises in children with sickle cell anemia. Pediatr Radiol.

[37] Frush DP, Heyneman LE, Ware RE, Bissett 3rd GS (1999). MR features of soft-tissue abnormalities due to acute marrow infarction in five children with sickle cell disease. AJR Am J Roentgenol.

[38] Jain R, Sawhney S, Rizvi SG (2008). Acute bone crises in sickle cell disease: the T1 fat-saturated sequence in differentiation of acute bone infarcts from acute osteomyelitis. Clin Radiol.

[39] Umans H, Haramati N, Flusser G (2000). The diagnostic role of gadolinium enhanced MRI in distinguishing between acute medullary bone infarct and osteomyelitis. Magn Reson Imaging.

[40] Ganguly A, Boswell W, Aniq H (2011). Musculoskeletal manifestations of sickle cell anaemia: a pictorial review. Anemia.

[41] Pineda C, Espinosa R, Pena A (2009). Radiographic imaging in osteomyelitis: the role of plain radiography, computed tomography, ultrasonography, magnetic resonance imaging, and scintigraphy. Semin Plast Surg.

[42] Lee YJ, Sadigh S, Mankad K, Kapse N, Rajeswaran G (2016). The imaging of osteomyelitis. Quant Imaging Med Surg.

[43] Roblot F, Besnier JM, Juhel L (2007). Optimal duration of antibiotic therapy in vertebral osteomyelitis. Semin Arthritis Rheum.

[44] Amundsen TR, Siegel MJ, Siegel BA (1984). Osteomyelitis and infarction in sickle cell hemoglobinopathies: differentiation by combined technetium and gallium scintigraphy. Radiology.

[45] Kim SK, Miller JH (2002). Natural history and distribution of bone and bone marrow infarction in sickle hemoglobinopathies. J Nucl Med.

[46] Berezin SW, Buchanan GR (1996). Differentiation of bone infarct from infection in a child in sickle cell disease. Pediatr Infect Dis J.

[47] Lutzker LG, Alavi A (1976). Bone and marrow imaging in sickle cell disease: diagnosis of infarction. Semin Nucl Med.

[48] Rao S, Solomon N, Miller S, Dunn E (1985). Scintigraphic differentiation of bone infarction from osteomyelitis in children with sickle cell disease. J Pediatr.

